# Terahertz Meets AI: The State of the Art

**DOI:** 10.3390/s23115034

**Published:** 2023-05-24

**Authors:** Arshad Farhad, Jae-Young Pyun

**Affiliations:** Department of Information and Communication Engineering, Chosun University, Gwangju 61452, Republic of Korea; arshad@chosun.ac.kr

**Keywords:** Terahertz (THz), artificial intelligence (AI), 6G, THz MAC protocols, 6G and beyond, THz simulators

## Abstract

Terahertz (THz) is a promising technology for future wireless communication networks, particularly for 6G and beyond. The ultra-wide THz band, ranging from 0.1 to 10 THz, can potentially address the limited capacity and scarcity of spectrum in current wireless systems such as 4G-LTE and 5G. Furthermore, it is expected to support advanced wireless applications requiring high data transmission and quality services, i.e., terabit-per-second backhaul systems, ultra-high-definition streaming, virtual/augmented reality, and high-bandwidth wireless communications. In recent years, artificial intelligence (AI) has been used mainly for resource management, spectrum allocation, modulation and bandwidth classification, interference mitigation, beamforming, and medium access control layer protocols to improve THz performance. This survey paper examines the use of AI in state-of-the-art THz communications, discussing the challenges, potentials, and shortcomings. Additionally, this survey discusses the available platforms, including commercial, testbeds, and publicly available simulators for THz communications. Finally, this survey provides future strategies for improving the existing THz simulators and using AI methods, including deep learning, federated learning, and reinforcement learning, to improve THz communications.

## 1. Introduction

5G mobile wireless communications have impacted transportation, healthcare, retail, finance, and manufacturing industries by providing high data rates and lower latency, leading to automation in industry and other businesses, and significantly increasing data traffic and intelligent device usage. 5G significantly improved data throughput, latency, network dependability, energy efficiency, and ultra-massive connectivity. Furthermore, the widespread use of smartphones, Internet of Things (IoT) devices, and new multimedia applications have significantly increased mobile data traffic. Therefore, it has revolutionized high-demand data applications such as video conferencing/streaming, virtual reality (VR), telemedicine, and online gaming.

**Figure 1:**
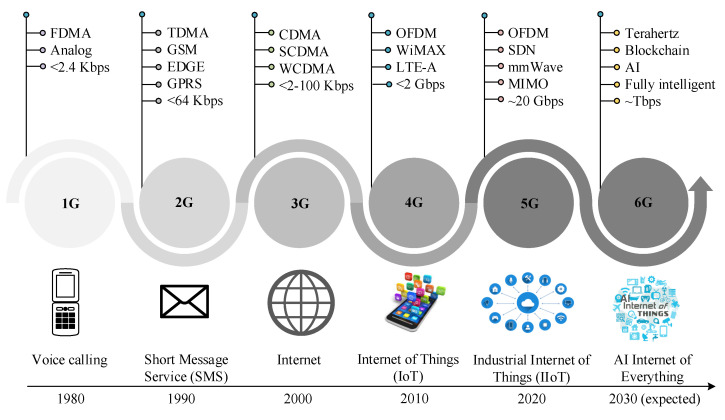
The progression of cellular networks, starting with 1G and advancing to 6G, has been accompanied by introducing various applications specific to each generation.

Figure 1 illustrates the continuous evolution of cellular wireless networks, where it can be seen that the evolution is primarily dependent on the increased traffic demands and network capacity. 1G cellular communication technology, mainly analog-based, was introduced in the 1980s, offering only basic voice communication with limited coverage and low capacity. 1G utilized frequency-division multiple access (FDMA) to divide the available spectrum into multiple channels, each of which could be used by a single user. The 2G, announced in the 1990s, offered voice calls and basic data services such as text messaging. In addition, 2G offered time-division multiple access (TDMA) to divide the available spectrum into time slots, improved voice quality, faster data speeds, and more features than 1G, such as a global system for mobile communications (GSM), general packet radio service (GPRS), enhanced data rates for GSM evolution (EDGE). 3G technology was launched in the 2000s, offering high-speed data services such as web browsing. 3G used code-division multiple access (CDMA), improving performance owing to the use of the same spectrum by multiple users. 4G was announced in the early 2010s, offering high-speed data services, such as video streaming and cloud gaming using orthogonal frequency-division multiplexing (OFDM). 5G technology was introduced in the 2020s to provide ultra-high data rates, lower latency, and massive device connectivity compared to 4G. In addition, 5G introduced millimeter-wave (mmWave), multiple-input and multiple-output (MIMO), beamforming [1], and network slicing, enabling faster downloads, more reliable connections, and support for IoT and smart cities. In addition, the standardization timeline of 5G with key features is illustrated in Table 1 [2,3,4,5]. In the next decade, the demands and needs will continue to grow owing to the diverse application requirements, leading to manifold new use cases where the requirements cannot be met by 5G networks, such as holographic teleportation, remote surgery, and unmanned aerial vehicles (UAVs).

As 5G begins to be widely adopted, research institutions worldwide have started to focus on developing 6G wireless communications, which is projected to be rolled out by 2030 [6]. For instance, the University of Oulu launched the 6G Flagship initiative at the beginning of 2018 to perform research activities and other things that could be important for the launch of 6G [7]. The 6G flagship initiative aims to investigate various use cases, including holograms, multi-sensory communications, Terahertz (THz) technology, and pervasive artificial intelligence (AI). Similarly, the US Federal Commission has also begun exploring the THz spectrum, issuing licenses to the University at Buffalo and the State University of New York to conduct outdoor experiments for developing improved propagation models at 240 GHz and 1.05 THz with effective radiated power (ERP) of 330 W.

### 1.1. Methodology

We began this survey with a systematic literature review methodology, as described in [8]. First, we started by looking for THz in conjunction with machine learning (ML)/deep learning (DL)/reinforcement learning (RL) in the abstract of each paper found on widely available databases. Then, we discarded out-of-scope papers and added some papers manually with backward reference searching and cross-citation techniques.

### 1.2. Scope of the Survey

Several surveys and tutorials have presented the THz communication technology and solution designed for 6G wireless communication systems in recent years. Table 2 focuses on and briefly describes the major topics addressed in the existing survey articles and tutorials currently available on THz communication technology. Mainly, these survey articles focus on antenna designs, medium access control (MAC) layers techniques, signal processing, spectrum allocation, collision analysis, resource management, modulation classification, interference mitigation, beamforming, and propagation attenuation, highlighting shortcomings and future strategies. In addition, the existing surveys in Table 2 focus on providing a comprehensive review of the THz communication and are not focusing on using ML methods for the THz performance improvements or discussing the available platforms such as commercial, testbeds and publicly available THz simulators. This survey fills this gap by presenting a constructive and comprehensive review of the use of AI in THz communications and available platforms. This survey also identifies issues and presents future recommendations to help researchers quickly identify the problem domain.

### 1.3. Contributions of the Survey

The contribution of this survey paper regarding existing surveys and tutorials is outlined as follows:

1. A systematic method to detail the different THz performance aspects that are improved using ML techniques. We will delve into key THz challenges and how they are addressed through ML applications.
2. This survey gives readers a general understanding of the state of the art and current trends in utilizing ML to address specific THz issues and challenges.
3. This survey provides an in-depth overview and analysis of the THz platforms, including commercial platforms, testbeds, and publicly available network simulators. Additionally, this survey presents future research prospects for advancing THz simulators and the application of ML to improve or evaluate THz performance and provides readers with an understanding of what still needs to be accomplished in THz using ML.

### 1.4. Structure of the Survey

**Figure 2:**
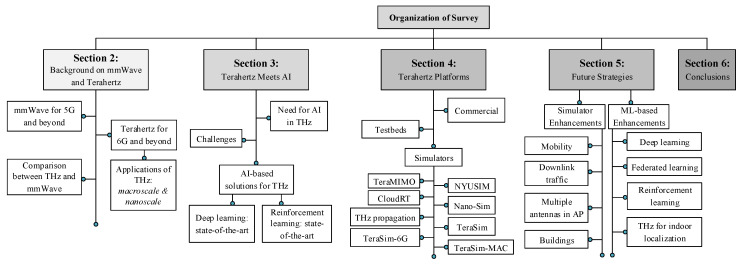
Structure of the survey, illustrating the hierarchical organization of the survey with the overall survey design.

Figure 2 illustrates the hierarchical structure of the survey, where Section 2 presents a detailed background on mmWave and THz along with THz applications and a comparative study of mmWave and THz. Section 3 presents the need for AI in THz, challenges, the state of the art using DL and RL with their potentials, and shortcomings. Section 4 provides an overview and analysis of THz platforms, including commercial, Testbeds, and publicly available network simulators. Section 5 suggests future strategies in improving THz communication using simulation and AI methods. Finally, the last Section 6 provides concluding remarks on this survey paper.

## 2. Background on mmWave and Terahertz

This section briefly discusses the mmWave and THz technologies and presents a comparative study of both technologies.

### 2.1. mmWave Overview

mmWave is a promising 5G mobile cellular network technology. Using mmWave MIMO small cells can significantly push the limits of legacy networks [40]. In the context of 5G and beyond, mmWave is expected to play a critical role in meeting the ever-increasing demand for data-intensive applications such as video streaming, real-time gaming, and virtual reality experiences [41]. The short wavelengths of mmWave signals also enable the deployment of small-cell wireless networks that provide high-speed connectivity in densely populated urban areas. The mmWave band consists of frequencies between 30 and 300 GHz, experiencing significant attenuation due to oxygen absorption [42,43]. However, 35, 94, 140, and 220 GHz frequencies have less attenuation and can be used for long-distance communication [42,44].

### 2.2. Terahertz Overview

The THz band, a trillion cycles per second, is the frequency range between 0.1 to 10 THz [12,45,46,47]. The advancement of wireless communication systems in the 6G and beyond is anticipated to rely heavily on THz technology [48]. With its ability to provide high-speed data transfer rates and low latency, THz technology is well-suited for various applications, including virtual and augmented reality [49], high-definition video streaming, and autonomous vehicles [33]. Higher data rates of up to Tbps make THz appropriate for imaging and communication networks. The THz band has been exploited for imaging for nearly two decades. Its non-ionizing properties make it safe for medical imaging and security checks. Furthermore, we discuss the standardization efforts on THz communications.

#### 2.2.1. Standardization Efforts on THz Communications

IEEE 802.15.3d is a standard for THz, which defines a MAC layer and physical layer (PHY) for switched point-to-point links. The MAC layer supports aggregation and block acknowledgment to improve efficiency at high data rates. The PHY layer operates in the sub-THz frequency range between 252 GHz and 325 GHz [50,51,52]. The IEEE 802.15.3d defines two PHY modes that can be used to transmit data at speeds of up to 100 gigabits per second (Gb/s) in the range between 2.16 GHz and 69.12 GHz.

The IEEE 802.15.3d had been developed for nine years, from 2008 to 2017 [50]. The IEEE 802.15.3 Task Group 3d, formed in May 2014, developed a standard for THz switched point-to-point connections and a standard for proximity links at 60 GHz. The THz switched point-to-point standard was approved and published in 2017. Furthermore, the development of the IEEE 802.15.3d standard is a significant effort, providing a foundation for developing new THz-based products and services, such as high-speed data links, medical imaging systems, and security and surveillance systems. The standard is also expected to accelerate the development of THz technology and its adoption by industry.

#### 2.2.2. Applications of THz

**Figure 3:**
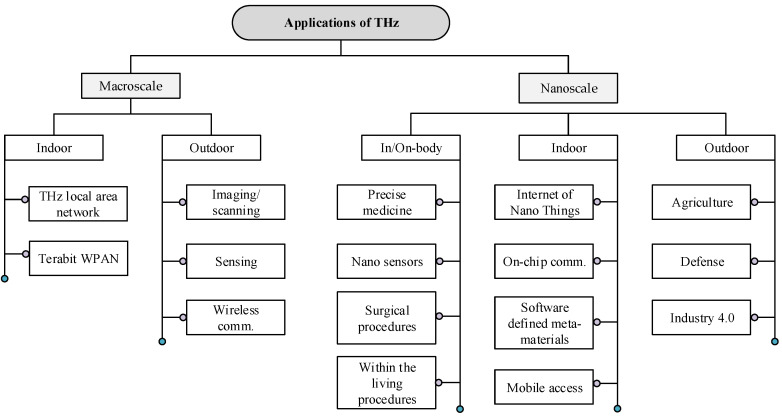
Terahertz band applications at the macroscale and nanoscale [12,56,57].

The applications of THz can be categorized into macroscale and nanoscale. These applications require high speeds, ranging from Gbps to Tbps, for outdoor and indoor [53]. Additionally, some applications require lower but still high speeds, such as Gbps, small-cell networks, wireless local area networks (WLAN) [54], and communication between vehicles and devices [55]. THz applications at the macroscale and nanoscale are highlighted in Figure 3 [56].

#### 2.2.3. Macroscale THz Applications

Macroscale applications range from 1–10 m [58] with dimensions greater than 100 nanometer (nm) [58]. Macroscale applications require Tbps links, including ultra-high-definition video and holographic conferencing, gaming, and VR. In conventional cellular networks, THz is suited for indoor applications or high-speed wireless backhaul small cells [57]. Similarly, in conventional WLAN applications, Terabit Wireless Local Area Networks (T-WLAN) can seamlessly connect high-speed wired networks, such as optical fiber links. In the same way, Terabit Wireless Personal Area Networks (T-WPAN) enable ultra-high-speed communication between nearby devices. For instance, kiosk downloading is one type of T-WPAN application where large video content is transferred to nearby devices [18,59]. Furthermore, high path loss and absorption (e.g., in indoor and outdoor scenarios) limit the distance of macroscale applications [60,61].

#### 2.2.4. Nanoscale THz Applications

Nanoscale applications are short-range, usually less than 1 m. Specifically, nanoscale refers to dimensions of 1 to 100 nm as defined in the “IEEE Standard Data Model for Nanoscale Communication Systems” and “IEEE Recommended Practice for Nanoscale and Molecular Communication Framework” [58,62]. The transmission distance of THz waves can vary depending on the application [18]. For example, THz waves can be used to transmit data over a few micrometers in medical imaging. However, it can also be used to transmit data over a few meters in the context of wireless communication [18].

A nanosensor network is formed by distributing several nanosensors around the body (e.g., for surgical procedures) for data collection, where every sensor is equipped with a carbon nanotube (CNT)-based nanoantenna, acting as a transceiver and communicates using the THz frequency band [58,63]. Nanotechnology allows the creation of such tiny, specialized nanocomponents that can perform specific tasks such as data storage, computation, and sensing [63,64,65]. These nanodevices can communicate with each other in a centralized or distributed manner, making them useful for biomedical, industrial, military, and health monitoring applications. As an example of nanoscale, sodium, glucose, and other ions in a body can be monitored [58]. THz communication can transmit the measured data from the human body to a device outside the body. These data can then be processed by an external device, such as a mobile phone or smart band, and sent to a medical device or doctor [18]. Other studies in [60,61] investigated the propagation in human tissues, focusing on fat absorption under the THz band. They revealed that fat absorbs THz waves strongly, limiting the range of communication between nanodevices in human tissues. However, the results also show that a distance of a few millimeters might be sufficient to ensure a reliable communication link.

### 2.3. Comparison between THz and mmWave

Table 3 briefly compares mmWave communication and THz band. The THz and mmWave bands are adjacent to each other, but they have distinct properties. For example, the THz band offers greater bandwidth than the mmWave band, allowing higher data rates and reduced interference [66,67,68,69].

The THz band has transmission windows that vary with distance, reaching up to THz bandwidth, while the mmWave band has a 10 GHz bandwidth and cannot achieve Tbps link speeds. For THz to attain data rates of up to 100 Gbps, the link capacity must be several times greater than the necessary user data rate to ensure prompt data delivery [59,116]. As frequencies increase in the THz band, Tbps links can achieve moderate and practical spectral efficiencies of a few bits per second per hertz. The THz band permits higher link directionality than mmWave at the same transmitter aperture due to less free-space diffraction and shorter wavelengths than mmWave. Smaller antennas with strong directivity in THz communications can also decrease transmitted power and interference. Additionally, the risk of eavesdropping is lower in THz bands than in mmWave bands because of THz high directionality beams [42,93,117,118].

## 3. Terahertz Meets AI

This section highlights state-of-the-art THz communications, including the need for AI in THz technology, challenges, state-of-the-art deep learning and reinforcement learning solutions, their potential, and shortcomings.

### 3.1. Need for AI in THz

AI comprises various techniques, such as ML, DL, and RL, that can be used to analyze and interpret large complex datasets. In recent years, the use of AI in THz has been gaining momentum as it is becoming increasingly important. Furthermore, the demand for faster and more efficient communication and sensing technologies continues to grow. Therefore, AI with reduced complexity is recommended in model and algorithm deficit cases [119]. The lack of a proper model is called the “model deficit,” which can be caused by a lack of knowledge about the specific domain.

On the other hand, the “algorithm deficit” refers to a situation where a mathematical model is appropriately known. However, the optimization of such mathematical models is considered challenging. Moreover, AI methods seamlessly integrate contextual data into the decision-making process. Thus, AI can optimize the performance of THz communication systems by adaptively adjusting the system parameters to account for changing channel conditions, THz sensing, advanced signal processing techniques, and so on [120].

### 3.2. Challenges

THz band has the potential to provide high data rates and low latency. However, it faces several challenges, including limiting the distance due to high path loss, propagation attenuation, and absorption in indoor and outdoor environments [60,61], the narrow bandwidth of the THz band [121], channel estimation and beam training in multi-hop THz communications for intelligent MAC design [98,122], resource allocation in D2D scenarios [123,124], and modulation schemes classifications. Researchers have used DL and RL methods to address these challenges, as discussed in Section 3.3 and Section 3.4, respectively. Additionally, the potentials and shortcomings of these methods are highlighted in Table 4 and Table 5.

### 3.3. Deep Learning: State of the Art

The emergence of DL in the THz is a cutting-edge and rapidly expanding field that holds tremendous promise for revolutionizing THz research and transforming its applications. Table 4 illustrates DL methods applied to improve the THz technology with their potential and shortcomings. The highlighted approaches in Table 4 are designed to improve beam prediction [125], overcome noise reduction [126], improve spectrum allocation [127], classify THz modulation [128], to analyze collision and overcome it by designing an intelligent MAC [98], identify the type of modulation used in THz communication system [129], improve resource allocation [124], and classify THz bandwidth [121].

The beam misalignment problem caused due to the mobility of UAVs [130], was investigated and resolved by the authors in [131] by proposing RNN based on echo state networks to reduce the outage probability of THz-band wireless links in UAV networks. Their proposed LeTera method uses a UAV mobility pattern to predict the optimal beam width. The LeTera was evaluated in various weather conditions under UAV flight experiments. The results of the LeTera method showed 99% accuracy, showing LeTera predicts the optimal beam width accurately. Furthermore, it was suggested that LeTera could be used to reduce outages and improve link capacity.

The paper [127] proposed an unsupervised learning-based approach for THz spectrum allocation in a multiuser THz network. Their proposed method divided the THz spectrum into sub-bands with unequal bandwidths to determine the optimal sub-bandwidth and transmit power. They trained a DNN model and approximated the near-optimal solutions. The numerical results illustrated that their proposed DNN method achieved a higher data rate than existing approaches.

The authors [128] proposed a convolutional neural network (CNN) approach, which uses the constellation diagram of the received signal as the input and applies a two-stage CNN architecture for modulation recognition. The first stage applies a convolution layer to extract the features, and the second stage uses fully connected layers for classification. The proposed approach was compared with other existing methods, and simulation results showed that it outperforms traditional techniques, especially for low signal-to-noise ratio (SNR) values.

**Table 4:** DL examples applied to THz technology.

Ref.	Year	DL Method	Potentials	Shortcomings
[131]	2020	RNN	Efficient beam alignment in UAVs	The LeTera scheme may not be effective in environments with obstacles since obstacles can block the beam and cause outages.
[125]	2021	GRU	Enhances beam prediction	It is dependent on a large training dataset for predicting the beamforming weights. It may limit the range and reliability of beam prediction even with advanced machine learning models due to the high attenuation of THz signals.
[126]	2021	DNN	Reduces noise	DNN model can result in poor performance when the model is applied to new THz signals with different types or levels of noise.
[127]	2022	DNN	Efficiently allocates spectrum	The performance of the DNN-based approach for spectrum allocation can be affected by the complexity of the wireless environment.
[128]	2022	CNN	Effectively classifies modulation	CNN is dependent on a large training dataset for accurately classifying different modulation schemes, which can be challenging to obtain in THz communication systems due to the limited availability of hardware.
[98]	2022	LSTM, GRU, and Bi-LSTM	Reduces collision of packets	The dataset collection is not described in detail, which limits the regeneration of the collision analysis through LSTM, Bi-LSTM, and GRU.
[129]	2022	CNN and LSTM	Recognizes signal modulation effectively	The approach may require large labeled data for training to achieve high accuracy, which could be a challenge to obtain in some indoor/outdoor scenarios.
[124]	2023	LSTM	Efficient resource management	Relies on simulation results rather than actual experimental data, which may limit the generalization of the findings to real-world scenarios.
[121]	2023	CNN	Classifies modulation and bandwidth	The experiments were conducted at a single frequency (120 GHz) and may not generalize to other frequencies in the THz band.
[65]	2023	CNN & GA	Efficient beam management at THz frequencies	Genetic algorithm is a heuristic algorithm, which may not guarantee the best solution.

**Table 5:** RL examples applied to THz technology.

Ref.	Year	DL method	Potentials	Shortcomings
[132]	2020	DRL	Reduces propagation attenuation	DRL requires large amounts of training data to learn the underlying propagation environment, which can be time-consuming and resource-intensive.
[133]	2020	RL	Mitigates interference	The proposed approach assumes that interference is sporadic and short-lived, which may not be the case in all scenarios and could limit the performance of the algorithm.
[134]	2021	MAB	Efficient resource allocation in D2D scenario under multi-hop communication	RL methods can be computationally expensive and may require significant resources to implement.
[135]	2022	FDRL	Improved throughput using beamforming	Only SINR feedback as CSI may not perform real-world scenarios, which could limit its practical implementation.
[136]	2022	DRL-based FL	Efficient resource allocation to D2D-enabled wireless networks	In a D2D environment, user mobility can vary widely. It may not conform to the assumed models, leading to sub-optimal resource allocation and reduced network efficiency.
[99]	2022	GAN	Improved routing in a dynamic network using intelligent MAC protocol	It does not account for interference caused by the movement of nodes in other communication links. This can result in unexpected disruptions in communication links and impact the overall network performance.
[137]	2022	DQN	Minimizes network latency efficiently	It does not account for collaborative computation offloading among multiple UAVs and potential errors in channel estimation.
[138]	2023	RL	Enhances throughput of the network	The work does not consider the impact of noise and interference on the performance of the routing scheme.
[38]	2023	DRL	Improved energy efficiency	DRL is a computationally intensive technique, which may not be suitable for large-scale UDNs.

The paper [129] proposed a DL method to recognize signal modulation schemes at the THz frequency band. First, the paper highlighted the challenges of base-band signal processing in the THz frequency band due to the erratic fluctuations caused by severe weather and urban multipath scattering. Next, the paper explored DL techniques to address this challenge, specifically CNN and long short-term memory (LSTM) networks, to recognize signal modulation schemes. Finally, their study established indoor and outdoor environments to test the proposed DL method in severe weather conditions and varying SNR situations. The results of the study demonstrated the efficacy of DL-based techniques for modulation recognition in the THz frequency band.

In [98], the authors enhanced the existing MAC protocol called Adaptive Directional Antenna Protocol for Terahertz (ADAPT) [115], using multi-layer recurrent neural network (RNN). The ADAPT protocol shown in [115] comprises a 3-way handshake scheme using a turning AP. After turning into a new sector, the AP transmits a “call to action (CTA)” control packet to nodes. The nodes answer with a “request to send (RTS)” packet to the AP if the node has data to send. The AP waits for $T_{wait}$ time. To this end, there are three possible cases: (1) zero RTS received, (2) 1 RTS received, and (3) greater than 1 RTS received. In case (1), the sector has no nodes; as a result, the AP skips the current sector and turns into a new sector. Meanwhile, in case (2), the AP has received exactly one RTS from the node. After $T_{wait}$ time, the AP sends “clear to send (CTS),” completing the 3-way handshake. After the 3-way handshake, data transmission occurs between the node and AP. Finally, in case (3), the AP has received more than 1 RTSs. In such a situation, the AP divides the transmission slot for nodes. However, only one data packet can be transmitted by the node to AP in each sector. The [98] analyzed the collision behavior of ADAPT protocol considering macroscale use case. In their simulation, the BS was placed in the middle of a cell comprising 18 m of radius, where the devices were distributed randomly (i.e., ranging from 15 to 960). Their study shows the collision rate of control frames for the two topologies (centered and uniform) and two population values considering 30 sectors and 960 mobile nodes. As the population density increases, the collision rate also increases. The [98] evaluated the efficiency of sector collision classification using different RNN types (LSTM, Bi-LSTM, and GRU). The test accuracy of sector collisions of Bi-LSTM was higher (i.e., 90.03%) compared to LSTM (85.04%) and GRU (i.e., 87.66%).

A new resource management scheme for THz communication is proposed in [124]. The scheme jointly optimizes the reflection coefficient of reconfigurable intelligent surfaces (RISs), the transmit power of the base station (BS), and the wideband THz resource block allocation. RISs are a type of metasurface that can be used to reflect and redirect electromagnetic waves. This can be used to improve the performance of THz communication systems by reducing path loss and increasing the bandwidth. The method in [124] adopts an LSTM relying on optimization and ensemble learning methods. Simulation results showed significant spectral efficiency gains for eMBB while ensuring the reliability and latency requirements of the URLLC service. The proposed scheme is a promising approach for improving the performance of THz communication systems. It can support various services, including eMBB, URLLC, and virtual reality/augmented reality (VR/AR). However, the real-time performance of the ensemble learning model is slightly worse than the optimization approach.

The authors in [121] leveraged a DL method based on CNN, aiming to classify modulation and bandwidth at the THz band. The dataset was collected at 120 GHz for various MCS and SNR. The CNN model was trained on the dataset and achieved accuracies of up to 78% and 90%. They also proposed a boosting technique to improve inference quality while accounting for memory and latency constraints and evaluated the latency of the proposed DL method by deploying it using FPGA. Their study provided critical insights into the potential of DL at the PHY layer for adaptive THz communications.

A new method for designing metasurfaces at THz frequencies was proposed in [65]. Their method combines CNN and genetic algorithms (GA). First, they trained a CNN to predict the amplitude and phase response of a metasurface based on the provided pattern, allowing it to speed up the forward prediction process. Then, once the CNN is trained, the authors create an inverse design model based on GA for the metasurface patterns that meet the desired amplitude and phase requirements. At the time of testing, it was shown that a metasurface pattern split the input beam into two beams with uniform power distribution. The GA-based metasurface design model found this pattern in only 10 min, faster than the traditional trial-and-error method.

### 3.4. Reinforcement Learning: State of the Art

RL has the potential to revolutionize THz technology by enabling more efficient and effective use of THz radiation in a wide range of applications. Table 5 illustrates RL methods applied to improve the THz technology with their potential and shortcomings. The highlighted approaches in Table 5 are designed to improve the propagation attenuation [132], interference mitigation [133], multi-hop communication [134], THz beamforming [135], resource allocation to D2D-enabled wireless networks [136], MAC protocol for LoS mobile networks [99], latency minimization [137] etc.

To address high propagation attenuation and molecular absorption, a hybrid beamforming scheme was proposed in a multi-hop environment [132]. They combined a digital beamforming matrix at the BS with analog beamforming matrices at the RIS. Their proposed method leverages DRL to optimize beamforming and combat signal loss. Simulation results showed that this approach increases the coverage range of THz communications by 50% compared to existing benchmarks.

The authors proposed an RL framework to mitigate intermittent interference in THz networks [133]. Their proposed RL framework uses an adaptive multi-thresholding strategy to mitigate interference from directional links in the time domain. Moreover, the approach does not depend on pre-existing knowledge of interference statistics, making it an appropriate solution for mitigating interference in ever-changing scenarios. Simulation outcomes validated the BER performance of the proposed technique when compared to conventional time-domain interference mitigation strategies.

To tackle the challenges associated with multi-hop THz communications, the authors [134] proposed a new approach to beam training based on RL. First, the authors of the paper divide the multi-hop THz link into several individual single-hop links. They then train an RL agent to dynamically and collaboratively select the number of beam training levels across all individual single-hop links. Their proposed RL solution, based on the multi-armed bandit (MAB) framework, demonstrated rapid convergence in simulations with random channels and noise. Furthermore, they showed that their approach achieves a significant performance gain over traditional hierarchical beam training with a fixed number of training levels. This new approach to beam training is a promising step towards realizing multi-hop THz communications. It is a more efficient and effective way to train beams and improve the performance of multi-hop THz links.

The paper [135] introduced federated learning (FL) approach to training the beam. First, the authors divided the cellular network into several base stations. They then trained a deep deterministic policy gradient (DDPG) agent at each BS to learn a THz beamforming policy with limited channel state information (CSI). Moreover, they update their DDPG models using hidden information to reduce inter-cell interference. The hidden information, which is interference from the estimated CSI, is extracted and exchanged by the federated edge learning (FEL) server. Simulation results indicated that their approach achieves a significant performance gain over conventional non-learning-based and existing non-FDRL benchmark optimization techniques. Additionally, simulation results indicated that the cellular network could achieve higher throughput when more THz CSI and hidden neurons of DDPG are incorporated.

The paper [136] proposed a resource allocation (e.g., mode selection, power control, and channel allocation) approach using DRL and FL. They then trained a DRL agent at each cell to learn how to allocate resources to maximize the overall capacity and minimize power consumption while guaranteeing the quality of service (QoS) requirement of both cellular and D2D users. The DRL agents are updated using FL, which allows them to learn from each other without sharing their data. Their simulation results indicated that the proposed approach is robust to channel variations.

In [99], the authors proposed a MAC algorithm for mobile THz airborne networks (TANs). Their proposed protocol comprises three distinct characteristics: spatiotemporal TAN state learning, precise two-tiered MAC operational control, and comprehensive TAN-specific MAC behavior management. The primary aim was to create a predictive network state estimation model using deep learning and generative adversarial networks (GANs), enabling the coordination of all one-hop neighbors for scheduled, antenna-aligned line-of-sight communication. In addition, the authors suggested implementing nested deep reinforcement learning (DRL) featuring outer and inner policy loops for determining high-level and low-level actions. Simulation results revealed the seamless, high-speed THz communication at the MAC layer, supported by robust RF links under mobility scenarios.

A DRL algorithm for joint optimization of UAV placement, resource allocation, and computation offloading is investigated [137]. The proposed deep Q-learning (DQN) and DDPG search for near-optimal solutions were studied in a highly dynamic environment. Simulation results in different scenarios demonstrated the effectiveness of the proposed algorithms in solving the formulated non-convex problem of minimizing latency. The proposed solution is a promising approach, increasing the system capacity and meeting the real-time demands of latency-sensitive applications.

An RL analytical model was proposed for a multi-hop scenario in [138], inspired by the human cardiovascular system based on the use of Markov decision processes (MDPs). Their proposed MDP model is a four-dimensional (4D), simultaneously characterizes the nanodevice motion in the bloodstream and its energy level. The model was evaluated using MATLAB and Simpy simulators considering a hand vein scenario. It was found that multi-hop scenarios can significantly improve the throughput of the nanonetwork without sharply penalizing other aspects, such as energy consumption. Furthermore, their study suggests that RL-based dynamic multi-hop routing schemes can be a valuable tool for flow-guided nanonetworks in the human cardiovascular system.

The authors in [38] proposed a decentralized DRL-based technique to maximize energy efficiency in ultra-dense networks. Their proposed DRL method comprises two networks: actor and critic. Actor-network is responsible for determining the node association using the local data. In contrast, a critic network determines the energy-efficient user association and informs the decision of the actor-network. In their proposed DRL method, the DRL agent in each base station can determine the user association decision. Their results showed that their method achieved significant energy efficiency gains (i.e., more than 50%) over conventional techniques. Furthermore, it was suggested that the method could be utilized for user scheduling and resource allocation.

## 4. Terahertz Platforms

To quickly progress in communication and networking solutions, it is essential to develop experimental testbeds and simulation tools in parallel [139,140,141,142,143,144]. The THz technology is rapidly evolving, and various commercial, testbed, and simulator platforms are available to researchers and developers. The remainder of this section discusses the available platform in detail.

### 4.1. THz Commercial Platforms

Typically, these platforms offer the most features and performance. Therefore, they are a good option for researchers and developers who need a turnkey solution that can be used to develop and deploy THz applications quickly and easily.

There are two types of commercial THz devices: time-domain spectroscopy (TDS) and photonic-based frequency-domain spectroscopy (FDS), a widely used method for THz measurements [71,72,73,74,145]. TDS devices use a THz pulse to excite a material and measure the time it takes for the material to respond. The time-domain response is then converted to a frequency-domain spectrum. TDS devices are non-destructive and can measure a wide range of materials. However, they can be time-consuming and require high-power THz sources. On the other hand, FDS is a continuous-wave and frequency-tunable method, producing narrow-band signals. These devices use a THz field to excite a material and measure the frequency-domain response of the material. FDS devices are fast and can measure a wide range of materials. However, they are sensitive to environmental factors and require high-quality optical components.

Various platforms are available from different vendors, offering different features and capabilities. As an example of THz platforms, such as ZEMAX THz Design Studio [146], TERAVIEW THz-3000 [147], Menlo Systems THz-QCL source [148], and Advantest THz-3000 [149] are available. A comparison table of these commercial platforms is illustrated in Table 6.

### 4.2. THz Testbeds

A testbed is a platform for testing and validating new technologies and techniques in a controlled and reproducible environment. Testbeds are essential for research and development in THz communication, allowing researchers to test and optimize the designs before deploying them in real-world scenarios. Recently, the TeraNova testbed was utilized in [143], offering a wide range of features, including point-to-point links, multi-hop networks, and free-space optical communications, making it ideal for the development and evaluation of new THz communication technologies. The TeraNova testbed is a joint project between the University of New South Wales in Australia and the University of California, Berkeley in the United States. The TeraNova platform is an integrated testbed for ultrabroadband wireless communications at THz frequencies [143]. The TeraNova testbed platform primarily comprises a transmitter and a receiver, operating at frequencies up to 1.05 THz. The transmitter is based on a Schottky-diode [150] frequency multiplying and mixing chain, and the receiver is based on a sub-harmonic frequency mixer. The transmitter and the receiver are integrated with digital signal processing back-ends that allow various modulation and coding schemes to be implemented.

### 4.3. THz Simulators

A simulator is an essential tool for analyzing and evaluating the performance of wireless communication systems. With the ever-increasing demand for high-speed and reliable communication networks, simulation tools have become even more critical in designing and optimizing these systems. In the context of THz communication, where experimental setups are often costly and challenging, simulations are even more valuable. Furthermore, simulation tools can help to validate and improve solutions by providing a safe and controlled environment. Here, we present several publicly available THz network simulators, TeraMIMO [108], NYUSIM [109,151,152], CloudRT [32,110], Nano-Sim [111], THz propagation [112], TeraSim [113], TeraSim-6G [114], and TeraSim-MAC [115].

#### 4.3.1. TeraMIMO

The TeraMIMO [108] channel simulator was designed in MATLAB for wideband ultra-massive MIMO THz communications. The simulator employs a Monte Carlo-based method to generate random channel realizations with realistic fading and shadowing effects by modeling the THz channel in both the time and frequency domains. It includes directional antenna patterns, beamforming, and spatial correlation features. TeraMIMO supports two modes of operations: (1) graphical user interface (GUI) and (2) MATLAB scripting. In the GUI mode, the user can input the simulation parameters (e.g., general, UM-MIMO, and THz-specific parameters) and then run the simulation. After the simulation is executed, the results are stored in the workspace. Overall, the TeraMIMO channel simulator is a powerful tool for researchers and engineers developing THz communication systems. It allows system performance evaluation in realistic channel conditions, and its modular design allows for the simulation of various scenarios. The ability to handle UM-MIMO systems and wideband signals makes it particularly useful for evaluating the potential of THz communications for future high-speed wireless networks.

#### 4.3.2. NYUSIM

The New York University (NYU) developed a simulator called NYUSIM based on their measurement for mmWave and THz channels [109,151]. The simulator was built on real-world measurements at multiple mmWave frequencies, from 28 to 73 GHz. NYUSIM 4.0 [152] has been expanded to the frequency range of 0.5–150 GHz and supports simulations for urban micro, urban macro, rural macro, and indoor hotspot scenarios [153].

#### 4.3.3. CloudRT

CloudRT is a cloud-based platform designed for ray tracing that can be used to simulate the performance of wireless communication systems, such as 5G and THz, in a variety of applications, such as vehicle-to-infrastructure (V2I) [32,110]. CloudRT supports various frequencies, from 450 MHz to 325 GHz. It has been used to simulate the propagation of THz waves in various environments, including urban, rural, indoor/outdoor, and railway [154,155,156,157,158]. The CloudRT is a powerful tool that can be used to design and optimize THz communication systems performance.

#### 4.3.4. Nano-Sim

Nano-Sim is an ns-3 extension developed by the authors of [111] to model electromagnetic nanoscale communication networks in the THz range with a propagation loss model adapted from [159]. However, the THz-band communication scenario is only implemented at the nanoscale. Moreover, it utilizes a streamlined channel model with the nano-node transmission range as its only parameter. Furthermore, it fails to account for the need for nanodevices for energy harvesting.

#### 4.3.5. THz Propagation

For frequencies up to 2 THz, the authors of [112] created a THz directional propagation loss model for ns-3. This model is used for the cases where both the transmitter and receiver use collimated lenses (e.g., light rays are parallel to each other), resulting in the signal in a minimum divergence. However, in the terahertz domain, atmospheric molecules cause signal attenuation. This module accurately predicts THz propagation loss for a single frequency. However, it is not a complete network simulator module since neither the PHY nor MAC layer is implemented.

#### 4.3.6. TeraSim

The Ultrabroadband Nanonetworking Laboratory (UN-Lab) developed ns-3 module for THz band, namely “TeraSim”. The simulator was designed for nanoscale and macroscale applications [113,115]. This ns-3 module can simulate THz-band communication networks, which operate in the frequency range of 0.1 to 10 THz [115,160]. TeraSim takes into account the unique characteristics of THz devices and the THz channel, including a standard channel module that uses a frequency-selective channel model, parallel modules for nanoscale and macroscale situations, a THz directional antenna model, and an energy harvesting model [161,162,163]. In addition, TeraSim implements PHY and MAC layers modules, tailored with well-known MAC protocols ALOHA and CSMA at the link layer.

#### 4.3.7. TeraSim-6G

TeraSim-6G was introduced in [114], which extends upon the previously developed TeraSim simulator [113]. The TeraSim-6G comprises two parts: MATLAB and ns-3. The TeraSim-6G implements the channel models from [82,161] in MATLAB. The MATLAB part is then integrated with the ns-3 module for a realistic simulation environment. The ns-3 part contains the mmWave and THz scripts for various scenarios, such as short and long distances, with coverage of 1 to 5 m and 10 to 20 m, respectively. TeraSim-6G considers a THz link operating at 1.0345 THz with 74 GHz of bandwidth and a mmWave link operating at 28 GHz with the maximum bandwidth allowed in 3GPP NR (e.g., 400 MHz) using a constant bit rate source with UDP at the transport layer.

#### 4.3.8. TeraSim-MAC

TeraSim-MAC [115] is the extended version of TeraSim ns-3 module [113], which describes the implementation of a new MAC protocol for the macroscale scenario. In addition, TeraSim-MAC models the peculiarities of the THz frequency-selective channel. In this module, the authors used IEEE 802.15.3d standard for high data rate wireless networks [50]. TeraSim-MAC uses a single carrier signal with a bandwidth of 69.12 GHz, operating in the frequency range of 252.72 to 321.84 GHz.

#### Comparative Analysis of the THz Simulators

A detailed comparison of the available THz ns-3 modules and other simulators is illustrated in Table 7. Among them, TeraSim is the widely adopted ns-3 simulator module in the literature due to open-source and the availability of basic THz features, including physical layer protocol (e.g., IEEE 802.15.3d) and a wide range of frequency spectrum. Apart from it, these two works [114,115] extended the TeraSim by adding exciting new THz features along the acknowledgment (ACK) support and MAC protocols.

## 5. Future Strategies

The future strategies are broadly categorized into simulator enhancements and using ML techniques to evaluate and potentially improve THz performance.

### 5.1. Simulator Enhancements

Current THz simulators offer a promising set of features, but they can be further enhanced to provide more realistic evaluations of the performance of THz systems. This can be achieved by adding mobility, downlink traffic, multiple antennas, and building environments. Therefore, it will allow the researchers to understand better how THz systems will perform in real-world scenarios, which will help to design more efficient and effective MAC protocols for THz system.

#### 5.1.1. Mobility

Mobility is one of the essential aspects of communication, where nodes can move from one location to another. In the 6G communications system, some applications require mobility in indoor and outdoor scenarios, for instance, tracking applications [55]. Thus, it would be beneficial to introduce node mobility in *TeraSim-MAC* ns-3 module to evaluate the behavior and performance of the THz system. The mobility models available in ns-3 include Constant Position, Constant Velocity, Constant Acceleration, Gaussian Markov, Hierarchical, Random Direction 2D, Random Walk 2D, Random Waypoint, Steady State Random Waypoint, and Waypoint [164]. The most common and widely adopted mobility model in the literature is the Random Walk 2D mobility model for asset tracking [165,166].

#### 5.1.2. Downlink Traffic

Downlink traffic refers to data transmission from a central source (access point or network server) to multiple receivers (mobile devices or other networked devices). THz communication poses challenges regarding signal propagation, as THz frequencies are affected by atmospheric absorption and scattering. Therefore, efficiently managing downlink traffic in a THz communication system will require advanced techniques such as beamforming [167], modulation, and error correction.

#### 5.1.3. Multiple Antennas in AP

Using multiple antennas in AP can significantly improve the performance of wireless communication systems. MIMO can be used to increase the capacity, coverage, and reliability of THz-based wireless networks. However, several issues must be addressed when implementing multiple antennas in THz APs. One of the main issues is the high cost of THz equipment, which can make it challenging to deploy large antennas. Additionally, the limited range of THz waves makes it difficult to achieve a wide area with a single AP.

AI-enabled solutions can be used to overcome these issues and optimize the performance of multiple antennas in THz APs. For example, AI-based beamforming techniques can direct the signal toward the intended user and reduce interference. Additionally, AI-based algorithms can be used to optimize the deployment of multiple antennas, considering factors such as the location of users, obstacles, and other environmental factors. AI-based techniques can also be used to dynamically adjust the transmission power and modulation scheme of the APs to adapt to changing channel conditions and improve the overall performance of the wireless network.

#### 5.1.4. Buildings

Building features can be introduced for a more realistic scenario for THz performance evaluation, such as scalability analysis in high-density urban areas, similar to [168]. NS-3 incorporates square-shaped buildings with dimensions and distances based on the Manhattan layout model, which includes correlated shadowing. Nodes within a specific building are classified as indoor, and their transmissions will encounter significant building penetration losses. The building parameters used for the urban environment in ns-3 are detailed in [169]. These parameters can be adopted in *TeraSim-MAC* to test the THz performance in a realistic environment.

### 5.2. ML-Based Enhancements

ML methods, such as DL, RL, and FL can be used to enhance the performance of THz in terms of interference, energy consumption, latency, and collision [170,171]. ML-based solutions will lead to the development of new THz systems that are more efficient, effective, and reliable.

#### 5.2.1. Deep Learning

THz research primarily concentrates on devices, channels, and the PHY layer. Only a few MAC protocols for the THz band have been surveyed in [16]. A recent study in [98] proposed a DL-enhanced MAC protocol for THz communications. The proposed protocol in [98] utilizes the TeraSim-MAC simulator module to improve the collision of data packets. The results of their study show that the proposed protocol can significantly reduce the number of collisions in THz networks, therefore improving the overall performance of the network. Furthermore, incorporating DL techniques would be valuable when designing MAC protocols for THz communications using the ns-3 module TeraSim-MAC [113]. DL can be used to learn the most active sectors, dynamically adjust beamwidth, and select the most efficient modulation and coding scheme (MCS). This will lead to significant performance improvements in THz networks in terms of energy consumption.

**Figure 4:**
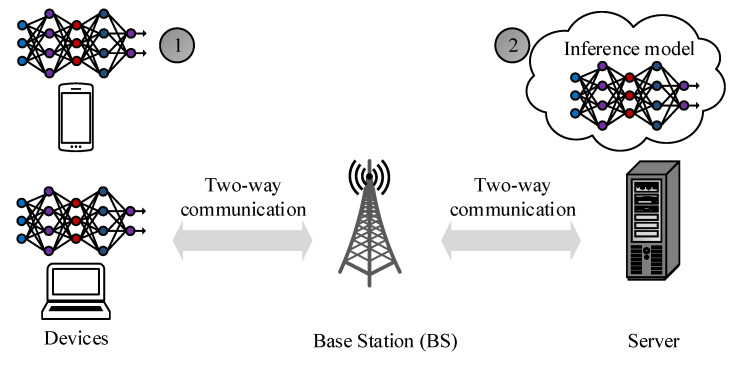
Example of centralized deep learning: in centralized deep learning, the ML model is trained on a centralized server using a one-time generated offline dataset. The trained model (i.e., pre-trained or inference model) is then deployed on the (1) devices or (2) the edge server for efficient resource allocation (e.g., transmit power and MCS).

A centralized DL approach is one example of using DL in the THz network. Generally, a centralized DL approach involves offline training and online observation processing for efficient radio resource allocation (e.g., MCS) to nodes [172,173]. The offline mode is mainly responsible for data collection (e.g., CSI features), cleaning, and training the DL model. After the DL model training, the pre-trained (i.e., inference) model is deployed on a network server or device, as illustrated in Figure 4. The inference model assigns resources based on the raw data generated during the network.

For instance, to use the inference model in ns-3, an open ecosystem called Open Neural Network Exchange (ONNX) [174] can be used as it is an open-source format for DL and traditional ML models. First, the ONNX-supported model can be generated using PyTorch. Then the pre-trained model (i.e., inference model) can be imported in ns-3 with the help of ONNX API for resource management by providing raw data during simulation.

#### 5.2.2. Federated Learning

**Figure 5:**
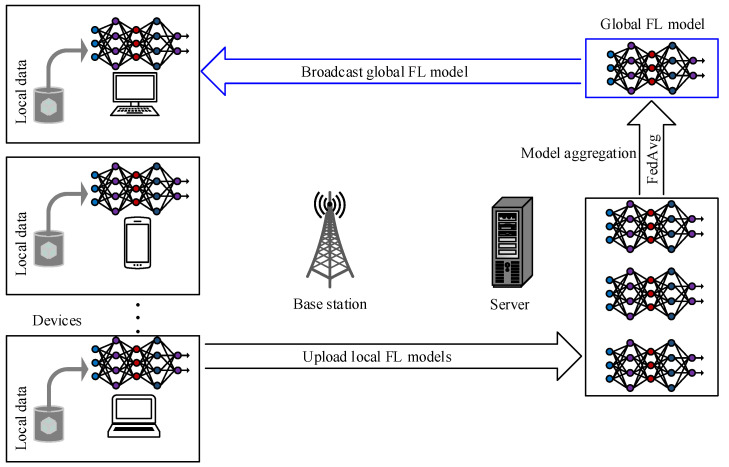
Example of federated learning: The global model is trained on a central server, and the local models are trained on the devices. The edge server and the devices share their models periodically to improve the global model.

FL is one the distributed machine learning methods that have been studied in various aspects of wireless communications [175,176,177,178]. Therefore, FL is considered one of the promising ML techniques for 6G communications [37,179,180,181,182], which can be utilized for THz in different ways, such as beamforming [38]. FL can be used to train a beamforming model optimized for the specific environment. This can improve signal strength and reduce interference between users. As an example, the authors in [183] have utilized FL for beamforming under ultra-dense mmWave networks. Therefore, the systematic design and performance analysis of beam management in FL-based ultra-dense THz networks is still open problems. Generally, FL trains an ML algorithm on decentralized devices without sharing the local data with a central server, as shown in Figure 5. The goal of FL is to improve the performance of ML models while protecting the privacy of user data.

For example, FL can be used in ns-3 using the ns-3-FL simulator [184,185]. The ns-3-FL simulator provides several features, including the FL algorithm, sharing global and local FL models between the devices and the central server, training the local models, and aggregating the local models to form a global model. Such features can be integrated with the TeraSim-MAC simulator to implement FL beamforming and modulation techniques.

#### 5.2.3. Reinforcement Learning

RL can enhance THz performance by learning from the interactions between the system and its surroundings. Applying RL for THz in ns-3 can provide a powerful tool for researchers and engineers to evaluate and optimize the performance of THz communication systems in a simulated environment.

One way to apply RL for THz in ns-3 is to optimize the parameters of a THz communication system, such as transmit power, modulation [186], and beamforming, based on the current channel conditions. For example, the RL algorithm can learn from past interactions with the simulated environment and adjust the system parameters to maximize a particular performance metric, such as data rate or capacity.

For instance, two widely adopted tools are available for RL in ns-3, such as ns3-gym [187,188] and ns3-AI [189,190].

**Figure 6:**
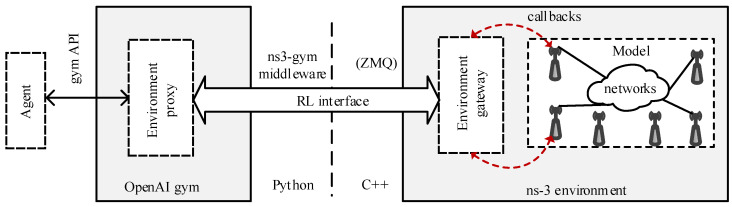
The architecture of ns3-gym utilized in ns-3 for RL [188].

**Figure 7:**
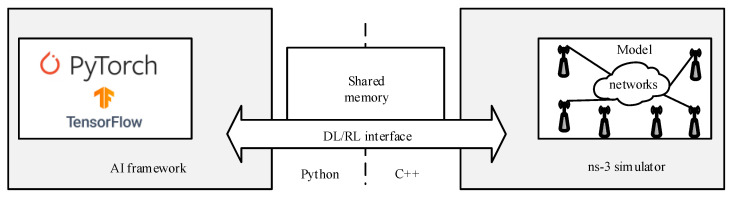
The architecture of ns3-AI utilized in ns-3 for reinforcement and DL [190].

1. *ns3-gym:* ns3-gym is a framework based on OpenAI Gym [191], allowing researchers from academia and developers from industry to use the ns-3 in conjunction with RL algorithms. Figure 6 illustrates the ns3-gym architecture comprising two primary components: the ns-3 and the OpenAI Gym framework. The former component is utilized for implementing environments, whereas the latter component is leveraged to unify the interface of these environments. Together, these components facilitate the development and deployment of robust and scalable RL algorithms for various networking applications. The authors in [188] utilized ns3-gym for radio channel selection in IEEE 802.11 network.
2. *ns3-AI:* The ns3-AI architecture is illustrated in Figure 7 [190], enabling data interaction between ns-3 and other Python-based AI frameworks (e.g., TensorFlow and PyTorch).
  The authors in [190] utilized the ns3-AI for (a) channel quality indicator (CQI) prediction in NR using LSTM and (b) TCP congestion control.

#### 5.2.4. THz for Indoor Localization

Current indoor localization systems rely on conventional wireless technologies such as Wi-Fi [192], Bluetooth Low Energy (BLE) [193], Ultra-Wideband (UWB) [194], Inertial Measurement Units (IMUs) [195], and Geomagnetic Field Based [196], which are often limited by low accuracy, high multipath propagation, and non-line-of-sight conditions. However, THz communication, which operates at a higher frequency (frequency range of 0.1 to 10 THz [12]) than conventional wireless technologies, has shown the potential to provide high accuracy with a mean distance error of 0.27 m [197] and 0.25 m [198].

The performance of these methods [197,198] can be further enhanced by implementing a multi-modal system, such as Wi-Fi, to generate a radio fingerprinting map with the THz technology. This will involve collecting RSSI data from the THz and Wi-Fi modules and processing it to create a more accurate radio fingerprinting map. Training the DNN model with the newly generated dataset from the multi-modal system and testing in the online phase for localization will involve using the trained DNN model to estimate the location of a mobile device based on the radio fingerprinting map generated from the THz and Wi-Fi modules.

## 6. Conclusions

THz is a promising technology for high-speed future wireless communication. However, many challenges need to be addressed before THz can be widely deployed, such as the high attenuation of THz signals in the atmosphere, beamforming, and medium access control layer protocols. One promising approach to overcoming these challenges is using AI, which can be used to develop new THz communication protocols that are more robust and efficient. Furthermore, the field of AI in THz is rapidly growing, with recent research and development focusing on various areas such as spectrum allocation, modulation and bandwidth classification, collision analysis, resource management, interference mitigation, intelligent beamforming, and intelligent medium access control layer protocols. Therefore, we expect more research and development, leading to new AI-based THz systems and applications.

In conclusion, this survey provides a detailed analysis of the state-of-the-art THz communications using AI. It discusses the available platforms, including commercial, testbeds, and publicly available network simulators. Finally, recommendations for THz are highlighted, which serve as a roadmap for improving the simulators and realizing the use of AI in THz.

## Figures and Tables

**Table 1:** 5G standardization timeline [3,4,5].

Release	Year	Key Features
15 (Phase 1)	2018	Non-standalone 5G new radio (NR), 5G core network, ultra-reliable low-latency communications (URLLC), evolved mobile broadband (eMBB), massive machine type communications (mMTC), beamforming, mmWave, long-term evolution (LTE)-NR dual connectivity, network slicing.
16 (Phase 2)	2020	Standalone 5G NR, enhanced URLLC, industrial IoT, NR sidelink with a focus on vehicle-to-everything (V2X), MIMO, NR-time sensitive networking (TSN) integration, network automation, NR positioning.
17 (Phase 3)	2022	5G NR evolution, enhanced MIMO, enhanced carrier aggregation, 5G NR in unlicensed spectrum, NR-based satellite communications, NR for private networks, AI/ML-based network automation and optimization, multi-access edge computing, NR-based device-to-device communication.
18 (Phase 4)	2024	Will improve physical random access channel coverage, latency, and signaling overhead in mobility scenarios.

**Table 2:** A summary of existing general surveys and tutorials on THz from 2019 to May 2023.

Ref.	Year	Primary Focus	Main Topics Covered by Surveys and Tutorials
[9]	2019	THz modulators	Reviewed THz modulators, evaluated devices, metamaterials, and modulation techniques for THz.
[10]	2019	THz channel features	Emphasized the important characteristics of THz channels and recent progress in device technologies.
[11]	2019	THz communications	Overviewed the advancements in THz communications and discussed the key technologies encountered in THz.
[12]	2019	THz channel models	Extensively presented the progress on THz device development and propagation model.
[13]	2019	THz-band operation	Surveyed the challenges, opportunities, potential applications, and future directions for THz.
[14]	2020	THz applications	Presented an outlook on THz and localization applications.
[15]	2020	THz channel features	Examined the characteristics of the THz channel and highlighted challenges.
[16]	2020	THz MAC protocols	Surveyed MAC protocols designed for THz.
[17]	2020	THz antennas analysis	Conducted a thorough examination of THz antennas, including their research background and fundamental principles.
[18]	2021	THz and mmWave	Comprehensively compared the mmWave and THz bands, and highlighted potential applications in 6G.
[19]	2021	THz capacity analysis	Presented characteristics and capacity analysis of THz channels.
[20]	2021	THz channel modeling	Focused on channel models and recent research efforts on factors affecting THz communication and discussed future research on THz channel modeling.
[21]	2021	THz technology potential	Examined various aspects of THz technology, discussed the potential, and researched THz technology towards commercialization.
[22]	2021	THz communications improvement	Examined intelligent reflecting surfaces (IRS) for improving THz communications and addressed active and passive beamforming challenges.
[23]	2021	THz communication	Presented an in-depth overview of how IRS and THz communications can work together to create a flexible and adaptable wireless communication environment.
[24]	2021	THz signal processing	Provided an in-depth overview of THz channels and summarized recent research efforts on modeling these channels, and highlighted challenges.
[25]	2021	THz for nanonetworks	Outlined areas where THz nanonetworks could be utilized, highlighting the specific requirements these applications would have for the supporting nanonetworks, which can be used as a guide for designing the necessary communication and networking protocols.
[26]	2021	DL techniques	Explored and discussed DL techniques for future 6G PHY communications systems.
[27]	2022	THz communication advancements	Summarized the advancements in THz communication over the past decade and identified the major obstacles that must be addressed for designing THz wireless systems in the future, including those for 6G networks, as well as short-distance connections and wireless connections for fixed locations.
[28]	2022	THz communication	Discussed THz communication aspects, examined theoretical approaches to analyze and design THz transmissions for increased connectivity and security, and explored spectrum management strategies.
[29]	2022	THz Propagation	Presented a comprehensive overview of the propagation channel characteristics and modeling for current THz applications.
[30]	2022	THz communication systems	Investigated the unaddressed issues and emerging research fields that require further exploration in the context of THz-band communication systems.
[31]	2022	THz-Empowered UAVs in 6G	Highlighted opportunities, challenges, and design strategies that influence the benefits of integrating THz-based features and UAV networks.
[32]	2022	THz channel characteristic	Provided in-depth review of THz channel characteristics, highlighted open issues, and future directions on THz.
[33]	2022	THz physical, link, and network layers	Covered THz spectrum management, antennas, and beamforming, as well as integrating other technologies that enable 6G in THz communication.
[34]	2022	THz antenna design	Provided an overview of how ML, FL, and AI are being used in antenna design.
[35]	2022	RL-based security and privacy	Reviewed reinforcement learning (RL)-based physical (PHY)-layer security techniques in THz.
[36]	2022	ML methods for THz	Investigated signal processing and ML methods for THz sensing.
[37]	2022	ML techniques	Presented the use of distributed ML for 6G networks including THz.
[38]	2023	ML for THz beamforming	Covered ML techniques applied to mmWave and THz for beam management.
[39]	2023	AI in THz for healthcare	Reviewed AI methods used to advance THz technology for cancer detection applications.

**Table 3:** Comparison between mmWave and THz wireless communication technologies [16].

Features	mmWave	Terahertz
Transceivers Device	Available [70]	Time-domain spectroscopy (TDS) and photonic-based frequency-domain spectroscopy (FDS) [71,72,73,74]
Modulation	High order modulation, supports 1024-QAM for downlink and 256-QAM for sub-7 GHz uplink [75,76,77]	low order modulation [57], supports Time Spread On-Off Keying (TS-OOK), Rate Division (RD-TS-OOK), Direct Sequence (DS-OOK) [78]
Antenna	Omni, directional, MIMO supported with high gain [79]	Omni and directional with phased array [75,80]
Bandwidth	0.03–0.3 THz [38]	0.1–10 THz [38]
Wavelength [mm] [55]	3–10	0.03–3
Array size [55]	10 × 10	100 × 100
Channel models [81]	Available [82,83,84,85,86,87]	Available [88,89,90,91,92,93]
Standards	5G NR [94], IEEE 802.11ad [95], and IEEE 802.11ay [96]	IEEE 802.15.3d [50]
Mobility	Yes [97]	Yes [98,99]
Beam Management	Yes [100]	Yes [38]
Outdoor Deployment	Yes [79,84]	Yes [90]
Free-space loss	Low	High
Coverage	High [101]	Low
Range	Up to 200 m or less [102,103]	Up to 10 m [18,57]
Device size	A few millimeters	1 to 100 nanometer [58,62]
Wavelength	1 mm–30 μm [104,105]	3 cm–1 mm
Data rate	Up to 10 Gbps [59,79]	Up to 100 Gbps [50]
Power consumption	Medium	Medium
Network topology	Centralized and clustered	Centralized, clustered, and distributed
Line-of-sight	Both	Both
Non-line-of-sight	Both	Both
Source of noise	Thermal and molecular noise	Thermal
Simulators	5G cellular network [106], 5G NR networks [107]	TeraMIMO [108], NYUSIM [109], CloudRT [110], Nano-Sim [111], THz propagation [112], TeraSim [113], TeraSim-6G [114], and TeraSim-MAC [115]

**Table 6:** Comparison of commercial platforms for THz communications considering various features.

Device	Manufacturer	Frequency Range	Wavelength Range	Applications
ZEMAX THz Design Studio	ZEMAX	0.1–10 THz	300 µm–3 mm	design and simulation of THz components and systems
TERAVIEW THz-3000	TeraView	0.1–3 THz	3 mm–100 µm	spectroscopy, imaging, sensing
Menlo Systems THz-QCL	Menlo Systems	0.1–10 THz	300 µm–3 mm	spectroscopy, imaging, sensing, communications
Advantest THz-3000	Advantest	1–10 THz	300 µm–3 mm	material inspection and analysis

**Table 7:** Comparison of available THz simulator modules and standalone simulators.

Features	Nano-Sim	THz Propagation	TeraSim	TeraSim-6G	TeraSim-MAC	TeraMIMO	NYUSIM	CloudRT
Simulation platform	ns-3	ns-3	ns-3	ns-3	ns-3	MATLAB	standalone	standalone
Application scenario	nano	nano	nano & macro	nano	nano & macro	macro	macro	macro
Physical layer protocol	✗	✗	✗	✗	IEEE 802.15.3d	✗	✗	✗
Propagation loss model	✓	✓	✓	✓	✓	✓	✗	✗
Energy harvesting model	✗	✗	✓	✓	✓	✗	✗	✗
Mobility	✗	✗	✗	✗	✗	✗	✗	✓
Adaptive MCS	✗	✗	✗	✗	✓	✗	✗	✗

## Data Availability

Not applicable.

## Abbreviations

The following abbreviations are used in this manuscript:

3GPP	3rd generation partnership project
ACK	Acknowledgment
ADAPT	Adaptive Directional Antenna Protocol for Terahertz
AP	Access Point
BER	Bit Error Rate
BLE	Bluetooth Low Energy
BS	Base Station
CDMA	Code-Division Multiple Access
CNN	Convolutional Neural Network
CQI	Channel Quality Indicator
CSI	Channel State Information
CTA	Call to Action
DDPG	Deep Deterministic Policy Gradient
DL	Deep Learning
DNN	Deep Neural Network
DQN	Deep Q-Learning
DRL	Deep Reinforcement Learning
EDGE	Enhanced Data Rates for GSM Evolution
eMBB	Evolved Mobile Broadband
ERP	Effective Radiated Power
FDMA	Frequency-Division Multiple Access
FDRL	Federated Deep Reinforcement Learning
FDS	Frequency-Domain Spectroscopy
GAN	Generative Adversarial Network
GPRS	General Packet Radio Service
GRU	Gated Recurrent Unit
GSM	Global System for Mobile Communications
IMUs	Inertial Measurement Units
InH	Indoor Hotspot
IoT	Internet of Things
IRS	Intelligent Reflecting Surfaces
ITU	International Telecommunication Union
LSTM	Long Short-Term Memory
LTE	Long-Term Evolution
MAC	Medium Access Control
MBC	Modulation and Bandwidth Classification
MCS	Modulation and Coding Scheme
MIMO	Multiple-Input and Multiple-Output
mMTC	Massive Machine Type Communications
mmWave	Millimeter-Wave
NR	New Radio
NYUSIM	New York University Simulator
OFDM	Orthogonal Frequency-Division Multiplexing
ONNX	Open Neural Network Exchange
PHY	Physical Layer
QoS	Quality of Service
RL	Reinforcement Learning
RMa	Rural Macro
RTS	Request to Send
TAN	THz Airborne Network
TCP	Transmission Control Protocol
TDMA	Time-Division Multiple Access
TDS	Time-Domain Spectroscopy
TSN	Time Sensitive Networking
UAV	Unmanned Aerial Vehicle
UMa	Urban Macro
UMi	Urban Micro
UN-Lab	Ultrabroadband Nanonetworking Laboratory
URLLC	Ultra-Reliable Low-Latency Communications
UWB	Ultra-Wideband
V2I	Vehicle-to-Infrastructure
V2X	Vehicle-to-Everything
VR	Virtual Reality
